# An IoT-Oriented Offloading Method with Privacy Preservation for Cloudlet-Enabled Wireless Metropolitan Area Networks

**DOI:** 10.3390/s18093030

**Published:** 2018-09-10

**Authors:** Zhanyang Xu, Renhao Gu, Tao Huang, Haolong Xiang, Xuyun Zhang, Lianyong Qi, Xiaolong Xu

**Affiliations:** 1 School of Computer and Software, Nanjing University of Information Science and Technology, Nanjing 210044, China; zhanyang_xu@nuist.edu.cn (Z.X.); renhaogu@gmail.com (R.G.); 2 Jiangsu Engineering Center of Network Monitoring, Nanjing University of Information Science and Technology, Nanjing 210044, China; 3 School of Computer Science and Technology, Silicon Lake College, Suzhou 215332, China; nuisthuangtao@163.com; 4 Department of Electrical and Computer Engineering, University of Auckland, Auckland 1023, New Zealand; hlx6700@gmail.com (H.X.); xuyun.zhang@auckland.ac.nz (X.Z.); 5 School of Information Science and Engineering, Qufu Normal University, Rizhao 276826, China

**Keywords:** IoT data, cloudlet, WMAN environment, privacy preservation

## Abstract

With the development of the Internet of Things (IoT) technology, a vast amount of the IoT data is generated by mobile applications from mobile devices. Cloudlets provide a paradigm that allows the mobile applications and the generated IoT data to be offloaded from the mobile devices to the cloudlets for processing and storage through the access points (APs) in the Wireless Metropolitan Area Networks (WMANs). Since most of the IoT data is relevant to personal privacy, it is necessary to pay attention to data transmission security. However, it is still a challenge to realize the goal of optimizing the data transmission time, energy consumption and resource utilization with the privacy preservation considered for the cloudlet-enabled WMAN. In this paper, an IoT-oriented offloading method, named IOM, with privacy preservation is proposed to solve this problem. The task-offloading strategy with privacy preservation in WMANs is analyzed and modeled as a constrained multi-objective optimization problem. Then, the Dijkstra algorithm is employed to evaluate the shortest path between APs in WMANs, and the nondominated sorting differential evolution algorithm (NSDE) is adopted to optimize the proposed multi-objective problem. Finally, the experimental results demonstrate that the proposed method is both effective and efficient.

## 1. Introduction

### 1.1. Background

A Wireless Metropolitan Area Network (WMAN) is a kind of mobile broadband wireless network, launched as a computer communication network within a city, which provides users with more convenient wireless services [1]. Metropolitan areas have high-density populations, where there are intensive data produced by the mobile devices in people’s daily lives. Mobile cloud computing provides a novel paradigm that allows the computing tasks and the data from the mobile devices to be offloaded to the remote cloud for processing and storage through access points (APs) in the WMAN [2]. With the increasing number of mobile devices and the rapid growth of mobile cloud computing technology, the WMAN draws a great development via its excellent features in transmission speed, coverage area and so on [3].

The rapid development of mobile and network technologies has resulted in the emergence of various intelligent mobile devices, including smartphones, tablets, Kindles, and so on [4]. However, the capacity of a mobile device is often limited by its battery, size and weight. Also, the mobile devices have fewer communication and computing resources compared with the desktop computers. Consequently, these devices are unable to perform the computing tasks that require high levels of processing and long working time. In order to solve this problem, mobile cloud computing technology is introduced, which migrates complex computing tasks for execution from the mobile devices to the cloud system [5,6]. In recent years, Internet of Things (IoT) technology has been developed rapidly, and puts forward improved requirements for task transmission delay [7,8].

Wearable technology, smart homes, smart medicals, smart car networks and so on are all parts of IoT technology which are connected to the sensors, controllers, machines, people and things via local networks or other communication technologies [9,10,11]. However, most mobile applications hosted in the mobile devices have vast data and high requirements for real-time processing [12]. Specifically, the distance between the cloud and the mobile devices is relatively long [13]. Therefore, the process of offloading may bring about high delay [14,15,16]. Such delay is intolerable for the mobile applications that require strict response time, such as HD live video, natural language processing and face recognition [17,18]. Thus, the cloudlet is hired to solve the above problems. Cloudlet is a cloud service enhancement technology which is composed by some small “clouds”, deployed on the edge of mobile devices [19].

Generally, there are many APs in the WMAN environment that are employed by the mobile users to access the cloudlets for efficient cloud services [20]. When the data in the mobile devices is transmitted to the cloudlet, it can be processed and the results can be returned efficiently [21]. However, with the increasing amount of IoT data, the problem of privacy leaks is getting worse [22]. Privacy preservation refers to the scenario where a cloudlet should not contain privacy conflicting information. When offloading the computing tasks with privacy conflicts on the same cloudlet, the privacy information of the mobile user, including home address, telephone and financial details, may be easily hacked [23]. Currently, some researchers use data encryption to deal with such security issues, but they were unable to deal with the troubles when the data were used legitimately [24].

On the other hand, the energy consumption and the transmission time are the important parts to evaluate the quality of IoT-oriented offload methods [25,26]. If the IoT data is encrypted, it will cost much more time in data processing and transmission, since the size of the data is greater. At the same time, more energy will be required for bigger data scales [27]. Considering the cloudlet environment under WMANs, the utilization of resources needs to be taken into consideration to avoid the overload or underload of the cloudlets. The resources in the cloudlet are provided in the form of virtual machines (VMs), which are assigned to multiple tasks elastically and on demand [28].

### 1.2. Motivation

Currently, a QoS (Quality of Service) aware cloudlet load balancing method for IoT data in WMAN is proposed [29], but it does not take into consideration the data privacy preservation. As a result, some important IoT data leak easily, causing great loss to users. Therefore, the privacy preservation of the IoT data in cloudlet-based WMAN environments has become an urgent problem. Hence, we consider a separated data offloading for the IoT data which has privacy conflicts. Meanwhile, an IoT-oriented offloading method (IOM) with privacy preservation is proposed to lower the transmission time, save energy consumption and improve the resource utility.

### 1.3. Paper Contributions

The main contributions of this paper include the following:

- Construct a systematic model of the resource utilization, the energy consumption and the data transmission time of the cloudlets when offloading the IoT data to the cloudlets.
- Adopt the Dijkstra algorithm to calculate the shortest path between AP points in a WMAN in order to reduce the transmission time of data.
- Optimize the multi-objective problem model by the nondominated sorting differential evolution (NSDE) algorithm with privacy preservation considered, and finally the optimal offloading strategies are output.
- Conduct extensive experimental evaluations and comparison analysis to demonstrate the efficiency and effectiveness of the proposed method.

The rest of the paper is organized as follows. Section 2 introduces the system model and the problem definition. Section 3 proposes an IoT-oriented offloading method with privacy preservation. In Section 4, simulation experiments and a comparison analysis are presented. Section 5 reviews related works. Finally, conclusions and future work are drawn in Section 6.

## 2. System Model and Problem Formulation

In this section, we present a system model that closely approximates the cloudlet environment in the WMAN first. Then, three optimized models of IoT data, that is, the transmission time model, the energy consumption model and the resource utilization model, are formed. Some key notations and descriptions used in the paper are listed in Table 1.

### 2.1. Resource Model

In this paper, we focus on the IoT-oriented data offloading with privacy preservation for the cloudlet-enabled WMAN. We consider a separated data offloading for the IoT data with privacy conflicts to optimize the transmission time, the energy consumption and the resource utilization. Suppose that there are *N* cloudlets, denoted as *C* = {*c*_1_, *c*_2_, …, *c_N_*} (each cloudlet has one host), which are deployed in the WMAN. There are *M* APs, denoted as *A* = {*a*_1_, *a*_2_, …, *a_M_*}. The cloudlets are connected through the APs, and *M* > *N*. *P* computing tasks that should be offloaded to the cloudlets for processing are donated as *T* = {*t*_1_, *t*_2_, …, *t_P_*}. The datasets of the computing tasks are donated as *D =* {*d*_1_, *d*_2_, …, *d_P_*}.

Let *X =* {*x*_1_, *x*_2_, …, *x_P_*} be the offloading policy for the IoT data of the computing task set *T*, where *x${}_{P\bar{I}}$* ∈ *C* (*p* = {1, 2, …, *P*}) is the cloudlet that the computing task *t_P_* is offloaded to.

**Figure 1:**
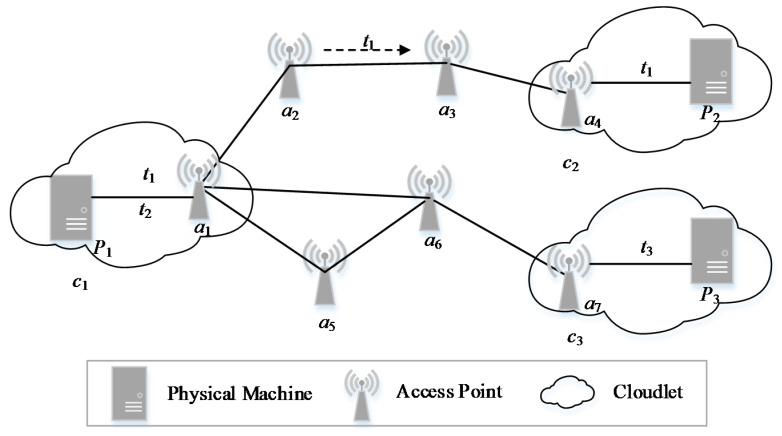
An example of cloudlet layout in the wireless metropolitan area network.

Figure 1 shows an example of cloudlet layout in the WMAN. The cloudlets are connected by the APs and deployed in the WMAN. There are seven APs, three physical machines, three cloudlets and three computing tasks in the example. The cloudlet *c*_1_ is connected to the cloudlet *c*_2_ through four APs, named *a*_1_, *a*_2_, *a*_3_ and *a*_4_. If the IoT data of the computing task *t*_1_ has privacy conflicts with the other data, *t*_1_ will be migrated to the cloudlet *c*_2_ for processing, through *a*_1_, *a*_2_, *a*_3_ and *a*_4_. In addition, the cloudlet *c*_1_ is connected to the cloudlet *c*_3_ through three APs, named *a*_1_, *a*_6_ and *a*_7_ or through APs of *a*_1_, *a*_5_, *a*_6_ and *a*_7_.

### 2.2. Resource Utilization Model

Compared to the mobile devices, the cloudlets have much greater physical resources including storage resources, computing resources and communication resources. When allocating the cloudlet resources to accommodate the IoT data, the resources are provided in the form of VMs. Assume that a cloudlet owns one physical machine. Let *h_n_* be the capacity of the *n*-th cloudlet *c_n_* and let *u_p,n_* be the requirements of the computing task *t_P_*.

The resource utilization is an important part to evaluate the efficiency of the cloudlets. According to the data offloading policy in X, the resource utilization of the cloudlet $c_{n}$ is $l_{n}\left(X\right)$, which is calculated by

(1) $$l_{n}(X)=\frac{1}{h_{n}}\sum_{p=1}^{P}u_{p,n}\cdot \theta_{p,n}(X),$$

where $\theta_{p,n}$ is a binary variable to judge whether $t_{p}$ is offloaded on $c_{n}$, which is measured by

(2) $$\theta_{p,n}(X)=\left\{ \begin{array}{l} 1,\;\mathrm{if}\;c_{n}=x_{p},\\ 0,\;\mathrm{Otherwise}. \end{array}\right.\;$$

Then, the number of the occupied hosts, denoted as σ(X), is calculated by

(3) $$\sigma (X)=\sum_{p=1}^{P}\sum_{n=1}^{N}\theta_{p,n}(X).$$

Finally, the average resource utilization of the cloudlet can be calculated by

(4) $$\psi (X)=\frac{1}{\sigma (X)}\sum_{n=1}^{N}l_{n}(X).$$

### 2.3. Data Transmission Model

The IoT data needs to be offloaded to the cloudlet for processing or the conflict data needs to be offloaded to another cloudlet. During the offloading, there is some transmission delay, which may influence the efficiency of the cloudlets. The transmission time for the IoT applications to offload datasets should be taken into account.

When the computing tasks are in the current cloudlet without offloading, the transmission delay is neglected. When the computing tasks are offloaded from one cloudlet to another, it will pass through multiple APs. Thus the transmission delay is added by the transmission time among APs.

Let *N_p,q_* be the number of halfway APs when the computing tasks are offloaded from the cloudlet *c_p_* to *c_q_*. Then, the transmission delay is calculated by

(5) $$TT(X)=\left\{ \begin{array}{l} 0,\;\mathrm{if}\;x_{p}=c_{n},\\ (2\cdot \frac{D_{p}}{\iota }+\frac{D_{p}}{\kappa }\cdot (N_{p,q}-1))\cdot \theta_{p,n}(X),\;\mathrm{Otherwise}, \end{array}\right.,$$

where *D_p_* is the data scale of *t_p_*. Then, the average data transmission time *T*(*X*) can be calculated by

(6) $$T(X)=\frac{1}{P}\cdot \sum_{p=1}^{P}\sum_{n=1}^{N}TT(X).$$

### 2.4. Energy Consumption Model

In this paper, the energy consumption is generated by the cloudlets, the active VMs and the idle VMs. The energy consumption is associated with the execution time of the computing tasks. Let $\beta_{P,n}\left(X\right)$ be the execution time of $x_{p}$ in $c_{n}$, and it can be calculated by

(7) $$\beta_{P,n}\left(X\right)=\frac{I_{p,n}}{\mu_{p,n}\cdot CPU_{n}},\;$$

where $I_{p,n}$ is the instruction length of $x_{p}$ in $c_{n}$, $\mu_{p,n}$ is the number of the occupied VMs $x_{p}$ in $c_{n}$, and $CPU_{n}$ is the running power of the physical machine in the cloudlet $c_{n}$.

The execution time of $c_{n}$ is denoted as ST(X), which is calculated by

(8) $$ST(X)=\overset{N}{\underset{n=1}{\max}}(\beta_{P,n}(X)\cdot \theta_{p,n}(X)).$$

During the tracked execution period ST(X), the energy consumption of the active VMs in the cloudlet is denoted as $E_{VM}^{active}\left(X\right)$, which is calculated by

(9) $$E_{VM}^{active}\left(X\right)=\overset{N}{\underset{n=1}{\sum }}\overset{P}{\underset{p=1}{\sum }}u_{p}\theta_{p,n}\left(X\right)\cdot ST\left(X\right)\cdot \eta ,\;$$

where η is the power rate for the running VM instances.

Before the data offloading, all the VMs are assumed as the idle VMs. When the IoT data are offloaded to the cloudlets for execution, some idle VMs are switched into active VMs. This switch also generates a certain amount of energy consumption. Hence the energy consumption of the initial idle VMs is denoted as $E_{VM}^{initial}\left(X\right)$, which is calculated by

(10) $$E_{VM}^{initial}\left(X\right)=\sum_{p=1}^{p}u_{p}\cdot \left(ST\left(X\right)-\beta_{P}\left(X\right)\right)\cdot \theta_{p,n}\left(X\right)\cdot \tau ,$$

where τ is the power rate for the idle VM instances.

The process by which an idle VM is changed into an active one also consumes some energy. The energy consumption to switch the situation of VMs is denoted as $E_{VM}^{switch}\left(X\right)$, which is calculated by

(11) $$E_{VM}^{switch}\left(X\right)=\left(h_{n}-\overset{P}{\underset{p=1}{\sum }}u_{p,n}\right)\cdot ST\left(X\right)\cdot \theta_{p,n}\left(X\right)\cdot \tau .$$

In this way, the energy consumption of the idle VMs in the cloudlet is denoted as $E_{VM}^{idle}\left(X\right)$, which is calculated by

(12) $$E_{VM}^{idle}\left(X\right)=\sum_{n=1}^{N}\left(E_{VM}^{initial}\left(X\right)+E_{VM}^{switch}\left(X\right)\right).$$

All the running cloudlets consume the baseline energy during the tracked execution period ST(X). Such baseline energy consumption for the cloudlets is denoted as $E_{C}\left(X\right)$, which can be calculated by

(13) $$E_{C}\left(X\right)=\sum_{n=1}^{N}\sum_{p=1}^{p}\theta_{p,n}\left(X\right)\cdot ST\left(X\right)\cdot \xi ,$$

where ξ is the power rate for the cloudlets.

Then, the total energy consumption denoted as E(X) is calculated by

(14) $$E\left(X\right)=E_{VM}^{active}\left(X\right)+E_{VM}^{idle}\left(X\right)+E_{C}\left(X\right).$$

### 2.5. Data Privacy Preservation Model of the Comouting Tasks

The IoT data contained in the computing tasks have different attributes, which may generate privacy conflicts. Putting the IoT data with privacy conflicts on the same cloudlet may make the IoT data be easily attacked by hackers and it will cause data leakage. Hence, the IoT data with privacy conflicts under this condition are offloaded to the other cloudlets for processing.

The privacy conflicts of the datasets are modeled by a graph G=(D,E), where D is the set of datasets and E represents the conflicting relations between two datasets in D. Additionally, $\left(d_{i},d_{j}\right)\in E$ represents that there is a privacy conflict between the datasets $d_{i}$ and $d_{j}$, which should be offloaded to different cloudlets for processing.

Then, the conflicting datasets of $d_{p}$ are denoted as $CD_{p},$ which is obtained by

(15) $$CD_{p}=\left\{d_{j}|\left(d_{p},d_{j}\right)\in E,p=\left\{1,2,\cdots ,P\right\}\right\}.$$

Hence, the conflicting collection is obtained by *X* $=\left\{x_{1},x_{2},\cdots ,x_{P}\right\}$, where *X*$=\left\{x_{i}|x_{i}\in CD_{p},i=\left\{1,2,\cdots ,\left|CD_{p}\right|\right\}\right\}$.

## 3. An IoT-Oriented Offloading Method with Privacy Preservation

In this section, we mainly encode the IoT-oriented offloading model with data privacy conflicts in the WMAN. We aim to maximize the resource utilization in (4), minimize the data transmission time in (6) and minimize the energy consumption in (14) while satisfying the privacy constraints in (15). The formalized multi-objective problem is optimized by NSDE, and the diversity and convergence of the population are ensured through the mutation and crossover operations. In the individual selection phase, NSDE uses the fast nondominated sorting approach and the crowded-comparison operator to ensure that individuals with the relatively best fitness values in the current population can be preserved for the next generation.

### 3.1. Shortest Path Acquisition of APs in WMAN Based on Dijkstra Algorithm

To estimate the transmission time among the APs, we adopt the Dijkstra algorithm to calculate the shortest path between the APs. In order to reduce the transmission time of IoT data, each AP selects the shortest path for transmission.

In Figure 1, all the computing tasks and the IoT data are uploaded to the APs closest to them and offloaded to the appropriate cloudlet for processing. However, in the WMAN, there may be multiple transmission paths between two APs. In order to reduce the transmission time of data, each AP selects the shortest path for transmission.

Assuming that the transmission rate between the APs is the same, the WMAN can be regarded as a set of undirected unweighted graphs, and each AP is a node on this graph. The Dijkstra algorithm is used to calculate the shortest path between the mobile nodes.

### 3.2. Optimization Problem Model by NSDE

The problem model proposed in this paper is summarized as a constrained multi-objective optimization problem. NSDE is an essential multi-objective optimization algorithm using real number coding whose mutation vector is generated by the parent difference vector and intersects with the parent individual vector to generate a new individual vector. In the parent population and the offspring population, the fast nondominated sorting approach and the crowded-comparison operator are performed, and the individuals with better target values are preserved for the next generation. Compared with other algorithms, it is more effective in solving the approximation of the global optimal solution set in multidimensional space.

In this section, the problem model is real-coded, performing crossover, mutation and selection operations, and the problem is using the fast nondominated sorting approach and the crowded-comparison operator in the selection phase to preserve the individuals with better fitness for the next generation, and through continuous iteration, it is constantly approaching the optimal solution set.

(1) Encoding: A distribution strategy for all the computing tasks uploaded from each AP point is represented by a chromosome, and each gene in the chromosome represents the execution location of the task, which means that the task will be assigned to the corresponding cloudlet for execution. Therefore, the range of values for each gene depends on the number of the cloudlets that are used to perform the computing tasks.

**Figure 2:**
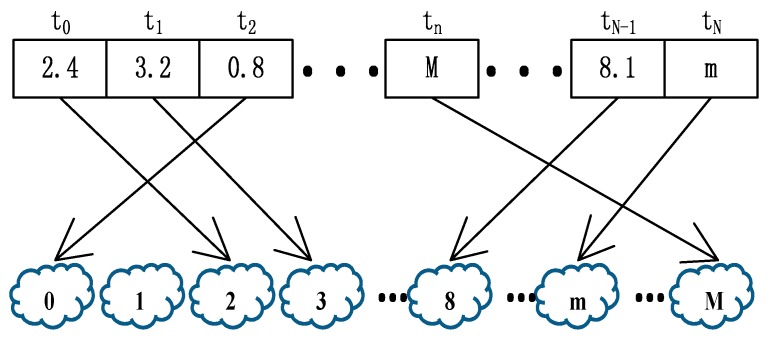
An encoding example of the computation offloading for the computing tasks.

Figure 2 shows the value of each gene in a chromosome. Suppose that the *n*-th task *t_n_* will be assigned to *M* cloudlets for execution. Then, the length of this chromosome *X_j_* is *N*, and each gene will be a real value between 0 and *M*. However, in the calculation, each real value will be converted into an integer that represents the position of the execution cloudlet. For example, in Figure 2, the second task *t*_1_ has a gene value of 3.2, and adopts the “down rounding” method, so the task *t*_1_ will be assigned to cloudlet 3 for execution.

(2) Fitness functions and constraints: A chromosome is an individual which represents an offloading strategy for all the computing tasks in the optimization problem. Multiple individuals constitute a population, and NSDE is used to optimize the population. The fitness functions are the criteria for evaluating each individual in the population, and the constraints are the conditions that each individual needs to satisfy during the problem optimization process.

There are three fitness functions for this optimization problem: the average resource utilization of the cloudlet, the data transmission time and the energy consumption, which are calculated by fitness functions (4), (6) and (14), respectively. In this optimization problem, the larger average resource utilization of the cloudlet with the smaller data transmission time and the lower energy consumption contributes to the better individual. Hence, the NSDE comprehensively evaluates all individuals in the population through these three fitness functions, not one or two of them.

However, during the evolution of the population, each individual also needs to meet two constraints: the total number of VMs requested by all the computing tasks on each cloudlet cannot be greater than the maximum number of VMs in the cloudlet, and the privacy preservation of the data is satisfied, which means the conflicting data cannot be offloaded to the same cloudlet.

(3) Initialization: Before the population is initialized, there are several algorithm parameters that need to be determined: the individual length *N*, which depends on the total number of computing tasks from all APs and each gene in the individual, represents the position of the cloudlet that the task is executed on, which has been introduced in the “Encoding” section; the size of population *NP*, which is usually set between *5N* and *10N*, but not less than *4N*; additionally, in the evolution of the NSDE, three parameters are used, including the crossover factor *CR*, the mutation factor *F* and the mutation strategy, where *CR* and *F* mainly determine the optimization ability and the convergence speed of the NSDE. The mutation strategy selected in this paper is “*DE/rand/1*”. After the parameters have been determined, the NSDE will generate a parent population *P* whose size is *NP* by initialization. The length of each individual is *N*, and the value of each gene in the individual is between *0* and *M*, where *N* and *M* represent the number of t computing tasks and cloudlets in the WMAN, respectively.

(4) Mutation and crossover: The mutation operation is performed by randomly selecting three individuals *X_a_*, *X_b_* and *X_c_* from the parent population *P*, and generating a mutated individual *H_i_* by combining the third individual *X_a_* with the difference vector of *X_b_* and *X_c_*, which is scaled according to the variation factor *F*. The crossover operation generates every gene *V_i,j_* of the offspring individual *V_i_* by crossing *X_i,j_* of the parent individual *X_i_* and *H_i,j_* of the mutated individual *H_i_*, where *X_i,j_*, *H_i,j_* and *V_i,j_* respectively represent the *j*-th gene of the parent individual *X_i_*, the mutated individual *H_i_* and the offspring individual *V_i_*.

(5) Selection: In the selection phase, based on the three fitness functions (4), (6) and (14), the NSDE performs the fast nondominated sorting approach and the crowded-comparison operator for the population O, which is composed of the parent population P and the offspring individual V_i_. The multiple nondominated layers *L_i_*(*i* = 0, 1, 2, …) will be generated by the fast nondominated sorting approach, and the individuals in the nondominated layer with the lower nondominated level or the individuals with a better crowding distance in the same nondominated layer are preferentially populated into the parent population *P* of the next generation until the size of the population *P* is exactly equal to *NP*. The method of crowding distance calculation is described as follows:

*D_j_* = *D_j_^U^* + *D_j_^T^* + *D_j_^E^* = |*U^j+^*^1^ − *U^j^*^−1^| + |*T^j^*^+1^ − *T^j^*^−1^| + |*E^j^*^+1^ − *E^j^*^−1^|, (16)

where *D_j_* represents the crowding distance, *D_j_^U^*, *D_j_^T^* and *D_j_^E^* represent the crowding distances of the average resource utilization, the data transmission time and the energy consumption, respectively. Besides, in (16), *U_j_*, *T_j_* and *E_j_* represent the objective function values of the average resource utilization, the data transmission time and the energy consumption, respectively, by the *j*-th offloading strategy *X_j_*.

(6) Iteration: The NSDE takes the population *P* generated by performing selection operations as the parent population of the next generation, and combines the population *P* and the mutation population *Q* generated by performing mutation and crossover operations into a population whose size is *2NP*. The parental population *P* of the next generation is regenerated by performing the selection operations on the population *O*. This process iterates until the termination condition is met, and finally the better solutions set *S* of the optimization problems is obtained.

NSDE-based migration strategy acquisition is illustrated in Algorithm 1.

**Algorithm 1.** nondominated sorting differential evolution based offloading strategy acquisition.**Input:** the size of the population *NP*, the number of the cloudlets *M*, the number of the iterations *G*, the mutation factor *F*, the crossover factor *CR* **Output:** the better solution set *S* 01: *g* = 0 02: *P* = Initialization (*NP*, *M*) 03: **while**
*g* < *G*
**do** 04:  *Q* = Crossover and mutation(*P*, *F*, *CR*) 05:  *O* = *P* + *Q*) 06:  *L* = nondominated sort(*O*)) 07:  *P = Ø*, *Q = Ø*, *i* = 0) 08:  **while** size(*P*) + num (*L_i_*) < *NP*
**do** 09:    *P* += *L_i_* 10:    *i* ++ 11:  **end while** 12:  Calculate crowding distance (*L_i_*) by (16) 13:  *L_i,j_ =* Sort (*L_i_*) according to *D_j_* from large to small 14:  *j = 0* 15:  **while** size(*P*) < *N*P **do** 16:    *P* += *L_i,j_* 17:    *j* ++ 18:  **end while** 19: *g* ++ 20: **end while** 21: **return** the better solution set *S*

## 4. Experimental Evaluation

In this section, a set of comprehensive simulations and experiments are conducted to evaluate the performance of the proposed IOM method. Specifically, we first introduce the simulation setup, including the simulation parameter settings and the statement of the comparative methods. Then, the influence of different task scales on the performance of the optimization metrics is evaluated.

### 4.1. Simulation Setup

In our simulation, three datasets with different scales of the computing tasks are applied for our experiments, and the number of computing tasks is set to 100, 150 and 200, respectively. The transmission speed of the cloudlets and the power rate of the cloudlets are set to 1200 M/s and 300 W according to [30]. The system decides which data items have conflicts according to the requirement of information security defined by the users or the processing records, which are assumed as the known information in our simulation. The specified parameter settings in this experiment are illustrated in Table 2.

To conduct the comparison analysis, we employ another basic offloaded method. The comparative method is briefly expounded as follows.

Benchmark: The task is offloaded to the nearest cloudlet first. If the task to be offloaded requires more resources than the current cloudlet owns or it has data conflicts with the computing tasks offloaded to the current cloudlet already, this task is offloaded to the cloudlet near the current one according to the Dijkstra algorithm. This process is repeated until all the computing tasks are offloaded to the cloudlets.

The methods are implemented under the simulation tools by CloudSim on a PC machine with two Intel Core i7-5500U 2.40 GHz processors and 8 GB RAM. The corresponding evaluation results are depicted in detail in the following sections.

### 4.2. Performance Evaluation of IOM

**Figure 3:**
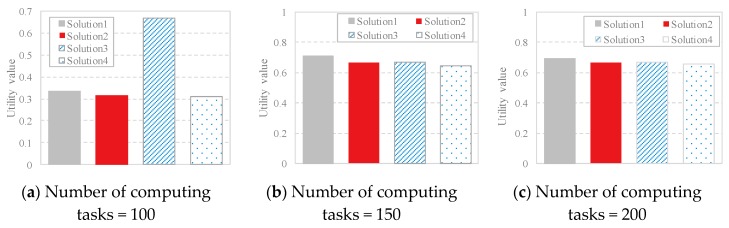
Comparison of the utility value of the solutions generated with different task scales by IOM.

The proposed IOM is intended to achieve a trade-off between optimizing the resource utilization, shortening the data transmission time and reducing the energy consumption while taking privacy preservation into consideration. We conducted 50 replicates of the experiment in the case of convergence for each task scale, and multiple sets of results were obtained. To identify a relatively optimal solution, simple additive weighting (SAW) and multiple-criteria decision making (MCDM) were used, where the optimal function is measured as follows:

(17) $$V\left(X\right)=\frac{1}{3}\cdot \frac{\psi \left(X\right)-\psi^{\min}}{\psi^{\max}-\psi^{\min}}+\frac{1}{3}\cdot \frac{T^{\max}-T\left(X\right)}{T^{\max}-T^{\min}}+\frac{1}{3}\cdot \frac{E^{\max}-E\left(X\right)}{E^{\max}-E^{\min}},$$

where Ψ(X), *T*(*X*) and *E*(*X*) represent the fitness of the data offloading strategy *x_i_* regarding the three objective functions mentioned above, respectively. Ψ^max^ and Ψ^min^ represent the maximum and minimum fitness values for the resource utilization. If Ψ^max^ = Ψ^min^, let $\frac{\Psi \left(X\right)-\Psi^{\min}}{\Psi^{\max}-\Psi^{\min}}=1$. Analogously, *T*^max^ and *T*^min^ represent the maximum and minimum fitness for the data transmission time, and if *T*^max^ = *T*^min^, let $\frac{T^{\max}-T\left(X\right)}{T^{\max}-T^{\min}}=1$; *E*^max^ and *E*^min^ represent the maximum and minimum fitness for the energy consumption, and if *E*^max^ = *E*^min^, let $\frac{E^{\max}-E\left(X\right)}{E^{\max}-E^{\min}}=1$. Figure 3 shows the comparison of the utility value of the solutions generated by IOM with different task scales. It is illustrated that when the task scale is 100, 150 or 200, four solutions are generated by IOM. For the solutions generated by IOM, we attempt to obtain the most balanced data offloading strategy by evaluating the utility value given in (17). After statistics and analysis, the solution with the maximum utility value is considered as the most balanced strategy. For instance, in Figure 3a, the final selected strategy is solution 3 because it achieves the highest utility value.

### 4.3. Comparison Analysis

**Figure 4:**
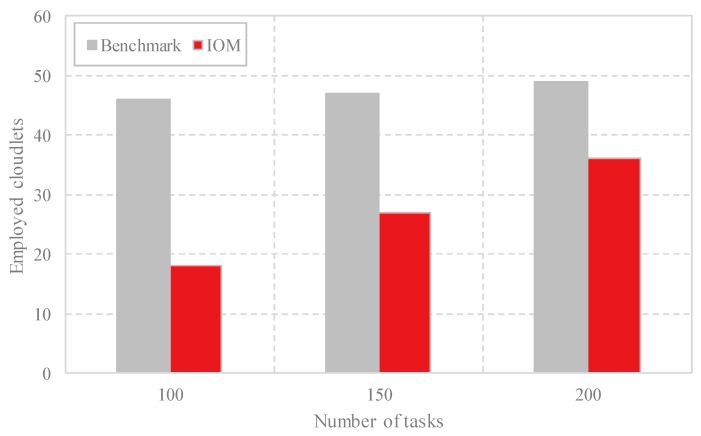
Comparison of the number of the employed cloudlets with different task scales by Benchmark and IOM.

**Figure 5:**
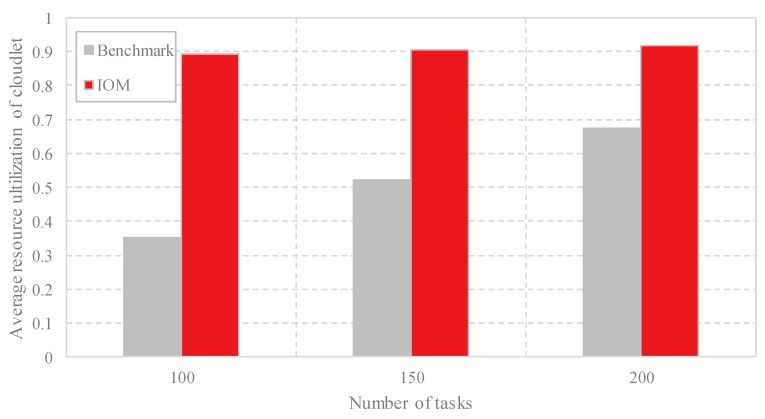
Comparison of the average resource utilization of the cloudlets with different task scales by Benchmark and IOM.

**Figure 6:**
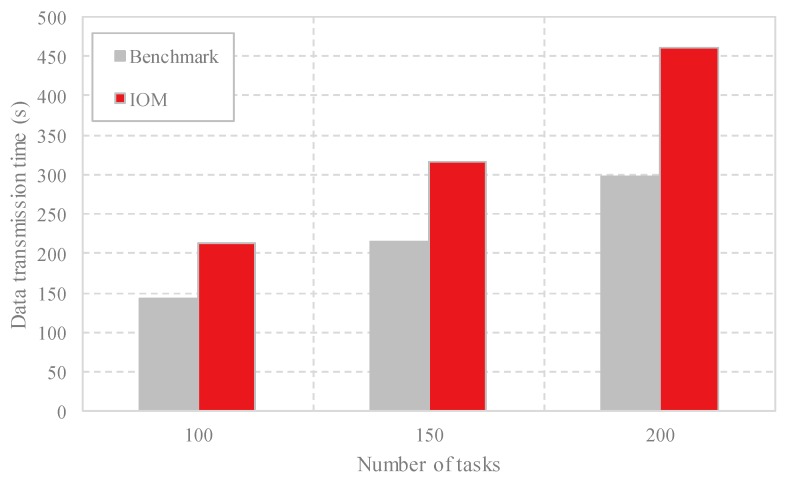
Comparison of the data transmission time with different task scales by Benchmark and IOM.

**Figure 7:**
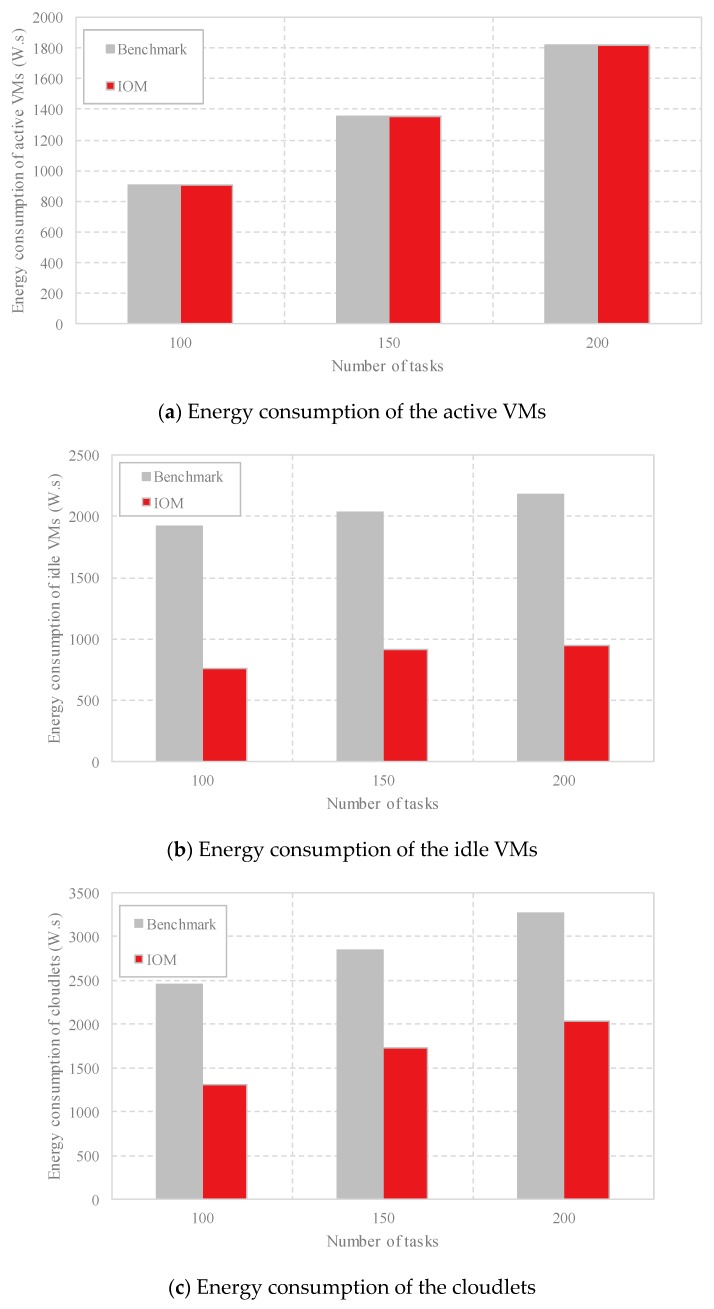
Comparison of the different components of the energy consumption with different task scales by Benchmark and IOM.

**Figure 8:**
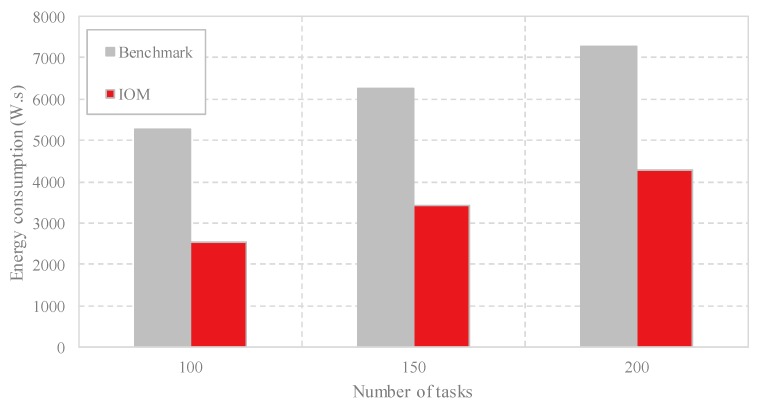
Comparison of the energy consumption with different task scales by Benchmark and IOM.

In this subsection, the comparisons of Benchmark and IOM with the same experimental context are analyzed in detail. The resource utilization, the data transmission time and the energy consumption are the main metrics for evaluating the performance of the data offloading methods. In addition, the number of employed cloudlets is presented to show the resource usage of all the cloudlets for offloading the computing tasks. The corresponding results are shown in Figure 4, Figure 5, Figure 6, Figure 7 and Figure 8.

(1) Comparison of the number of employed cloudlets: Figure 4 illustrates the number of cloudlets employed by the data offloading methods. The total number of the cloudlets in our experiment is set to 50. As shown in Figure 4, IOM employs fewer cloudlets compared with Benchmark. Furthermore, as the number of the computing tasks increases, the number of the cloudlets employed by IOM increases.

(2) Comparison of the resource utilization: After offloading all the computing tasks to the cloudlets via the data offloading methods, the occupation of the VMs is achieved. Figure 5 shows the comparison of the resource utilization of the cloudlets by using Benchmark and IOM with different task scales. The resource utilization is calculated according to the number of occupied cloudlets and the employed VMs in each cloudlet. Fewer employed cloudlets with more employed VMs contribute to a higher resource utilization. It is intuitive from Figure 5 that IOM achieves higher and stable resource utilization. That is, IOM reduces the number of unemployed VMs and wastes less resources than Benchmark.

(3) Comparison of the data transmission time: In Figure 6, we compare the data transmission time of the different data offloading methods. It is intuitive that our proposed method IOM costs more time than Benchmark. With the increase of the task scales, the data transmission time is enlarged. This may be because our proposed method needs more transmission times to realize the goal of optimizing the resource utilization and the energy consumption, which may sacrifice some transmission time on the other hand.

(4) Comparison of the energy consumption: As outlined in Section 2, the energy consumption is composed of the energy consumption of the active VMs, the energy consumption of the idle VMs, and the energy consumption of the cloudlets. In Figure 7, we compare these three aspects respectively with different task scales. As shown in Figure 7a, both methods achieve the same energy consumption of the active VMs at the same task scale because the same number of VMs are employed by Benchmark and IOM. Figure 7b shows that as the number of computing tasks increases, both methods increase the energy consumption of the idle VMs, but IOM generates less energy of the idle VMs due to less unemployed VMs used compared with Benchmark by occupying fewer cloudlets. Figure 7c indicates that IOM consumes less energy of the cloudlets than the Benchmark. The comparison of energy consumption in Figure 8 shows that IOM has better performance. For example, when the number of computing tasks is 100, IOM achieves a power consumption of less than 3000 W.s, whereas Benchmark generates more than 5000 W.s energy.

## 5. Related Work

With the development of the IoT technology, more IoT data is produced by mobile devices in daily life. Edge cloud computing developed rapidly to solve the transmission delay of the IoT data, providing high-speed processing in cloud service [31]. One of the hot technologies of edge cloud computing is the cloudlet, which is applied to get a shorter response time and reduce the energy consumption of mobile devices by alternating the offloading destinations, compared to the traditional mobile cloud computing paradigm [32,33,34,35]. There have been many studies about cloudlets, which were fully investigated in [36,37,38,39,40,41,42], to name a few.

In [36], the author studied the placement of the cloudlets in a large WMAN, consisting of many wireless APs. In order to realize the resource sharing of mobile users, Hoang et al. [37] used a cloudlet as a semi-Markov decision process (SMDP) to formalize a dynamic optimization problem. The SMDP is converted into a linear programming model to get the best solution. In the optimization model, mobile users need to consider different types of service quality under resource constraints. In [38], the author proposes a Performance-Enhancement Framework of Cloudlet (PEFC) to enhance the service performance of a cloudlet with limited resources. That paper aims to enhance the performance of the cloudlet and improve the experience of cloud service with limited resources. Artail et al. [39] proposed a general solution based on a mobile intelligent device to solve the service delay of the remote cloud. The author considered a cloud network, which distributes within a region and connects to the root server, to ensure resource availability. The framework is applicable to the environment where the cloudlet clients can sense networks and software services. Ciobanu et al. [40] introduced the drop computing paradigm, which proposes the concept of decentralized computing over multilayered networks, combining cloud and wireless technologies over a social crowd formed between mobile and edge devices. Mao et al. [41] jointly optimized task offload scheduling and transmission power allocation for mobile edge computing systems to reduce execution latency and device power consumption. The author proposed a low-complexity suboptimal algorithm to minimize the weighted sum of execution delay and device energy consumption, based on alternating minimization. Although the research on cloudlets is increasing, people often overlook the optimization of resource utilization, transmission delay and energy consumption when taking the privacy protection into account [43,44,45,46].

Current research mainly focuses on the capacitated cloudlets’ placement to save energy or encrypt data to prevent data leakage. In [47], the author studied the cloudlet placement and mobile user allocation to the cloudlets in the WMAN. The author also designed a cloudlet placement algorithm, which placed the cloudlet in a user-intensive area of the wireless metropolitan area networks to balance the workload of WMAN. Mahadev et al. [48] introduced GigaSight, which is an internet-scale crowdsourced video content repository with powerful privacy preferences and access control features. The GigaSight architecture is a joint system of VM-based cloudlets that performs video analytics on the edge of the internet, reducing the need for cloudlet ingress bandwidth. Rahman et al. [49] proposed a mobile edge computing framework that provides real-time and location-aware personalized services for a large number of users. According to the new privacy policy paradigm, it can make a secure share of location. The framework uses server-side cloud blending and crowd edge fog computing terminals (FCTs) to switch tasks between FCTs and the cloud, based on network condition, geographic location and available resources. Chen et al. [50] used the flexibility of the cloudlets to create a novel healthcare system. Cloudlet features include privacy protection, data sharing and intrusion detection. In the data collection phase, the author used the Number Theory Research Unit (NTRU) method to encrypt data collected by wearable devices. Then, they proposed a new trust model to help users choose trusted partners to share stored data in the cloudlet and help similar patients to communicate with each other. Finally, in order to protect the medical system from malicious attacks, the author developed a new collaborative intrusion detection system (IDS) method based on cloud networks.

Generally speaking, researchers do not take into consideration the data privacy preservation when optimizing the energy consumption of the cloudlets in the WMAN, or ameliorate the transmission time, energy consumption and resource utility when encrypting data [51,52,53]. Thus, an IoT-oriented offloading method with privacy preservation is proposed in this paper to optimize the transmission time, the energy consumption and the resource utilization when considering data privacy preservation.

## 6. Conclusions and Future Work

With the rapid development of IoT technology, the computing tasks of mobile applications have become so complex that it is necessary to offload the computing tasks to the remote cloud. For some applications with low latency requirements, it is necessary to offload the computing tasks to the nearby cloudlets for execution. Meanwhile, we have to be considerate of data conflicts to realize privacy preservation. In order to tackle such problems that have happened in the cloudlet-based WMAN environment, an IoT-oriented offloading method with privacy preservation is proposed in this paper to optimize the transmission time, the energy consumption and the resource utilization. Concretely, the task-offloading strategy with privacy preservation in the WMAN is modeled as a constrained multi-objective optimization problem. In order to reduce the transmission time, the Dijkstra algorithm is adopted to calculate the shortest path among APs in the WMAN. The multi-objective optimization problem is solved by an NSDE algorithm, and finally the best task-offloading strategy in the WMAN is obtained.

In future work, we will attempt to adapt and extend our proposed method to a real-world scenario for cloudlet services in the WMAN environment. Additionally, the privacy preservation strategy will be updated on the basis of the IoT data. At the same time, more attributes of the real-world scenario will be added to confirm the accuracy of our experiment.

## Figures and Tables

**Table 1:** Key notations and descriptions.

Terms	Descriptions
*C*	The cloudlet collection
*A*	The AP(Access Point) collection
*T*	The computing task collection
*D*	The dataset collection of the computing tasks
*X*	The data offloading policy collection for *T*
*P*	The number of computing tasks
*l_n_*(*X*)	The resource utilization rate of the cloudlet *c_n_*
*σ*(*X*)	The number of the occupied cloudlets
*ψ*(*X*)	The average of *l_n_*(*X*)
*TT*(*X*)	The propagation delay time of the computing tasks
*T*(*X*)	The average of propagation delay time
*β_P_*_,*n*_(*X*)	The execution time of the task *x_p_* in the cloudlet *c_n_*
*ST*(*X*)	The maximal execution time of the task in the cloudlet *c_n_*
$E_{VM}^{idle}\left(X\right)$	The energy consumption of the idle VMs (Virtual Machines)
$E_{VM}^{active}\left(X\right)$	The energy consumption of the active VMs
*E_c_*(*X*)	The energy consumption of the cloudlets
*E*(*X*)	The total energy consumption

**Table 2:** Parameter settings.

Parameter Description	Value
The total number of cloudlets	50
The maximum number of VMs a cloudlet owns	10
The transmission speed of AP	540 M/s
The transmission speed of the cloudlet	1200 M/s
The execution speed of the VMs	2000 MHz
The power rate of the active VMs	50 W
The power rate of the idle VMs	30 W
The power rate of the cloudlets	300 W
